# *trt-1* Is the Caenorhabditis elegans Catalytic Subunit of Telomerase

**DOI:** 10.1371/journal.pgen.0020018

**Published:** 2006-02-10

**Authors:** Bettina Meier, Iuval Clejan, Yan Liu, Mia Lowden, Anton Gartner, Jonathan Hodgkin, Shawn Ahmed

**Affiliations:** 1 Department of Genetics, University of North Carolina, Chapel Hill, North Carolina, United States of America; 2 Wellcome Trust Biocenter, University of Dundee, Dundee, United Kingdom; 3 Lineberger Comprehensive Cancer Center, University of North Carolina, Chapel Hill, North Carolina, United States of America; 4 Department of Biology, University of North Carolina, Chapel Hill, North Carolina, United States of America; 5 Genetics Unit, Department of Biochemistry, University of Oxford, Oxford, United Kingdom; Stanford University, United States of America

## Abstract

Mutants of *trt-1,* the Caenorhabditis elegans telomerase reverse transcriptase, reproduce normally for several generations but eventually become sterile as a consequence of telomere erosion and end-to-end chromosome fusions. Telomere erosion and uncapping do not cause an increase in apoptosis in the germlines of *trt-1* mutants. Instead, late-generation *trt-1* mutants display chromosome segregation defects that are likely to be the direct cause of sterility. *trt-1* functions in the same telomere replication pathway as *mrt-2,* a component of the Rad9/Rad1/Hus1 (9–1–1) proliferating cell nuclear antigen–like sliding clamp. Thus, the 9–1–1 complex may be required for telomerase to act at chromosome ends in *C. elegans.* Although telomere erosion limits replicative life span in human somatic cells, neither *trt-1* nor telomere shortening affects postmitotic aging in *C. elegans.* These findings illustrate effects of telomere dysfunction in C. elegans mutants lacking the catalytic subunit of telomerase, *trt-1.*

## Synopsis

In the 1930s, the maize geneticist Barbara McClintock observed that broken chromosome ends occasionally became stable when transmitted through the germline, and she inferred that a protective cap present at normal chromosome ends could be added to broken chromosomes de novo. We now know that short, repetitive DNA sequences cap chromosome ends in most eukaryotes and that such sequences can be added de novo by the telomerase reverse transcriptase, which uses an RNA template for telomere repeat addition. The authors genetically define the reverse transcriptase subunit of telomerase in the roundworm Caenorhabditis elegans. Proteins that respond to abnormal DNA damage are required for telomere replication in *C. elegans,* suggesting that chromosome ends may be recognized as a special form of DNA damage prior to telomere repeat addition by telomerase. The authors found that telomerase and DNA damage response proteins appear to function in the same telomere replication pathway. Replicative aging in human somatic cells may be caused by telomere shortening. However, somatic cells do not proliferate in C. elegans adults, and telomere erosion does not affect the aging process in this context. Finally, the authors observed that chromosome mis-segregation may explain the progressive sterility of C. elegans telomerase mutants.

## Introduction

Telomeres in most organisms are composed of simple repetitive sequences whose length is maintained by the telomerase ribonucleoprotein, which reverse transcribes telomere repeat sequences onto chromosome ends. Two subunits of telomerase are sufficient for generating telomerase activity in vitro: the telomerase reverse transcriptase (TERT) and the telomerase RNA, which contains the template sequence used by TERT for telomere repeat addition [1–3]. In the absence of telomerase, the DNA replication machinery is unable to completely replicate the chromosome terminus and telomere attrition occurs [4].

Most normal human somatic cells are deficient for TERT and therefore experience progressive telomere shortening when proliferating [1,2,5]. Telomere erosion in human primary cells grown in vitro typically causes replicative senescence within 40 to 60 population doublings [6]. Telomere erosion is exacerbated in patients with Werner's progeria (a segmental aging syndrome), and the in vitro premature senescence phenotype of Werner's cells can be overcome by expression of TERT [7]. Further, shortened life span and decreased cellular proliferative capacity are observed for late-generation mouse telomerase RNA mutants [8], and mice deficient for both the telomerase RNA and the Werner helicase display a variety of premature aging phenotypes in only a few generations [9,10]. These results provide compelling evidence that telomere erosion may affect proliferative aging in humans.

The nematode Caenorhabditis elegans was selected to study the impact of telomerase and telomere length in an organismal context. C. elegans has telomeres composed of simple TTAGGC repeats that are 2 to 4 kilobases in length [11]. The first C. elegans telomere replication mutant to be identified, *mortal germline-2 (mrt-2),* was recovered in a genetic screen for *mortal germline* mutants that are initially fertile but become progressively sterile if passaged for multiple generations [12]. *mrt-2* mutants become sterile as a consequence of progressive telomere shortening and end-to-end chromosome fusions. In addition, *mrt-2* mutants are hypersensitive to ionizing radiation, and *mrt-2* encodes an ortholog of a subunit of the 9–1–1 proliferating cell nuclear antigen (PCNA)–like sliding clamp that is loaded onto aberrant DNA structures at sites of genome damage [12]. Further evidence that the 9–1–1 DNA damage response complex is fully required for telomere replication in C. elegans is provided by HUS-1, another subunit of the 9–1–1 PCNA-like heterotrimer that physically interacts with MRT-2 [13]. Mutation of *hus-1* also results in telomere erosion, end-to-end chromosome fusions, and late-onset sterility [14]. Here we report the identification of the C. elegans catalytic subunit of telomerase, *trt-1,* and several consequences of telomere dysfunction in strains deficient for *trt-1.*

## Results

### Identification of the C. elegans Telomerase Reverse Transcriptase

**Figure 1:**
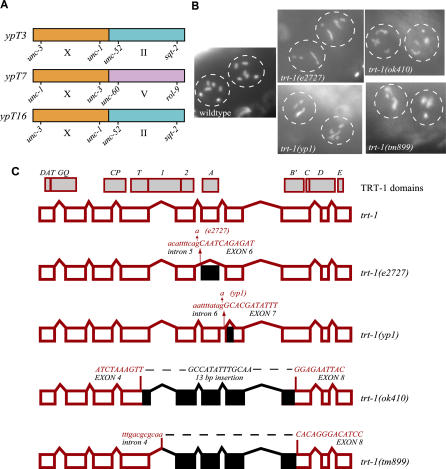
*trt-1* Is the C. elegans Catalytic Subunit of Telomerase (A) Three X-autosome fusions isolated from *trt-1(e2727)* display linkage between an end of the X chromosome and an end of an autosome. *ypT3* and *ypT16* were independent chromosome fusions isolated from parallel lines grown from the same F2 founder, suggesting that the two fusiogenic chromosome ends may correspond to short telomeres in the F2 founder, recapitulating our previous observations regarding strains inheriting a short left end of the X chromosome in *mrt-2* mutants [12] and supporting a mouse study of telomere length and genome instability in the absence of the telomerase RNA [61]. (B) End-to-end fusions in oocytes of late-generation *trt-1* mutant worms. Two DAPI-stained oocyte nuclei are shown per picture as indicated by dashed circles. (C) *trt-1* gene structure with mutations. Exon-intron structures and TERT protein domains are shown. Effects of mutations for all four *trt-1* alleles are shown as determined by genomic DNA and cDNA sequencing. Black boxes are used to depict exons missing as a consequence of *trt-1* splice-junction mutations or sequences eliminated by *trt-1* deletions. For *yp1,* intron 6 is retained and a cryptic intron in exon 7 becomes active. Sequences flanking each breakpoint are shown for *trt-1* deletions. A 13-bp in-frame insertion occurred at the breakpoint of *trt-1(ok410)*. Sequencing of cDNA from *trt-1(tm899)* indicated that the deletion results in a premature stop codon.

Based on previous observations with *mrt-2* and *hus-1* [12,14], continuous propagation of C. elegans telomere replication mutants was expected to result in uncapping and fusion of de-protected chromosome ends as a consequence of telomere erosion. C. elegans end-to-end chromosome fusions produce a dominant chromosome loss phenotype when heterozygous [12]. A small pilot screen of 200 ethylmethane sulfonate–mutagenized F2 lines yielded a single *mortal germline* mutation, *e2727,* that displayed a dominant Him (high incidence of males or X-chromosome nondisjunction) phenotype when late-generation *e2727* hermaphrodites were crossed with N2 wild-type males. Genetic mapping showed tight linkage of the dominant Him phenotype to one end of the X chromosome and to an end of an autosome in each case (Figure 1A), indicating the presence of X-autosome end-to-end chromosome fusions. These chromosome fusions were viable when homozygous, suggesting that their breakpoints were close to chromosome termini (unpublished data). Further, microscopy of late-generation *e2727* hermaphrodites revealed fewer than the normal complement of six bivalents in oocytes, the only C. elegans cells that arrest in metaphase and are therefore amenable to quantification of chromosome number, confirming the presence of end-to-end chromosome fusions (Figure 1B). Southern analysis of telomere length in *e2727* indicated that this mutant displayed progressive telomere shortening (Figure S1). The Mortal Germline phenotype of *e2727* was used to map this mutation to a genetic location of +3 on Chromosome I (six of nine Unc-non-Dpy recombinants from + *e2727 + */ *unc-13* + *dpy-24* F1 heterozygotes carried *e2727* and unpublished data). BLAST analysis identified a nearby gene, DY3.4, with weak but significant homology to telomerase reverse transcriptases. Sequencing of DY3.4 in the *e2727* mutant revealed a splice-acceptor mutation for intron 5 (Figure 1C), which resulted in mis-splicing and nonsense-mediated degradation of 90% of the DY3.4 message in *e2727* (unpublished data), as judged by RT-PCR analysis in a *smg-2* background, which is defective for nonsense-mediated mRNA decay [15]. However, 10% of the DY3.4 message is still spliced correctly in *smg-2,e2727* doubles, as commonly occurs for C. elegans splice-junction mutations.

A second allele of DY3.4, *yp1,* was identified in a large-scale screen for *mortal germline* mutants as a telomere replication mutant that mapped to the center of Chromosome I (Y. Liu and S. Ahmed, unpublished data). Two additional alleles of DY3.4, *ok410* and *tm899,* were recovered by groups searching for deletions of DY3.4 in pools of mutagenized worms. Consistent with the *e2727* mutant phenotypes, *yp1, ok410,* and *tm899* strains initially had normal brood sizes but displayed late-onset sterility over successive generations. A reduced chromosome complement was observed for late-generation strains near sterility (Figure 1B) but not for early or middle generations (unpublished data). Further, a dominant Him phenotype was observed for F1 derived from late-generation *yp1, ok410,* and *tm899* hermaphrodites crossed with wild-type males (unpublished data). Genetic mapping of the dominant Him phenotype from a number of independent *ok410* strains revealed tight linkage to one end of the X chromosome and to the end of an autosome in each case (M. Lowden and S. Ahmed, unpublished data), confirming that end-to-end chromosome fusions had been isolated. As *yp1, ok410,* and *tm899* appeared to have telomere replication defects caused by mutations in or near the DY3.4 gene, complementation tests were conducted. The *e2727, yp1,* and *tm899* mutations all failed to complement *ok410,* but not a tightly linked mutation in *unc-29,* for the late-onset sterility phenotype (unpublished data), indicating that *e2727, yp1, tm899,* and *ok410* are allelic.

**Figure 2:**
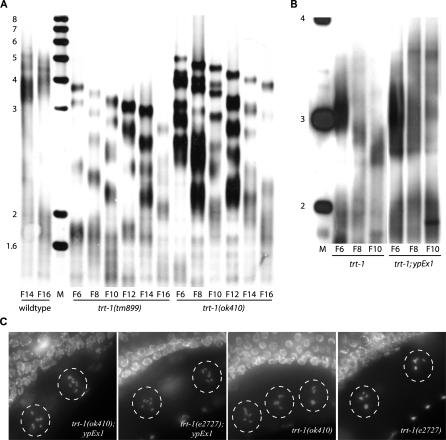
Progressive Telomere Shortening in *trt-1* Mutants and Rescue by the Wild-Type DY3.4 Gene (A) The *trt-1* alleles *tm899* and *ok410* displayed comparable rates of telomere shortening over progressive generations. Two generations of N2 wild-type are shown for comparison. Southern blotting was performed using a (TTAGGC)_n_ probe and genomic DNA prepared from propagated strains as described [12]. (B and C) Expression of DY3.4 on the *ypEx1* extrachromosomal array (B) eliminated telomere shortening for *trt-1(ok410)* in comparison with a nonrescued control and (C) repressed the formation of chromosome fusions observed in late-generation *ok410* and *e2727* mutants. Adult animals were stained with DAPI at generation F10, where nonrescued siblings exhibited a strongly reduced brood size or sterility. For Southern blots, sizes of DNA fragments are indicated to the left (kb).

The DY3.4 gene was sequenced in *yp1* and found to contain a G-to-A splice acceptor mutation for intron 6, which results in retention of this intron and generation of a premature stop codon at the intron-exon boundary. The DY3.4 deletions *ok410* and *tm899* eliminate coding sequence for several essential reverse transcriptase motifs of the DY3.4 protein and therefore ought to be null alleles (Figure 1C). Southern blotting of genomic DNA prepared from *trt-1* mutants revealed comparable rates of telomere erosion for *ok410* (125 ± 17.5 base pairs [bp]/generation), *tm899* (128.8 ± 27.5 bp/generation), *yp1* (111.3 ± 18.3 bp/generation), and *e2727* (123.6 ± 16.5 bp/generation) (Figures 2A and S1), despite the fact that *e2727* expressed some correctly spliced DY3.4 messenger RNA. The Mortal Germline phenotypes of *e2727* and *ok410* were rescued by extrachromosomal arrays carrying the wild-type DY3.4 gene, as nonrescued siblings of two *e2727* and one *ok410* strain became sterile within nine or ten generations postinjection, whereas their rescued siblings were still fertile after 32, 22, and 17 generations, respectively. Rescue of *ok410* was transient, probably as a consequence of progressive silencing of the rescuing transgene, as is commonly observed for transgenes in the C. elegans germline. Analysis of telomere length by Southern blotting revealed that the telomere shortening phenotypes of *e2727* and *ok410* were also rescued by the wild-type DY3.4 gene, which conferred the diffuse telomeres expected from stochastic telomere repeat addition by telomerase (Figure 2B and unpublished data). Moreover, staining of oocyte nuclei with 4′,6-diamidino-2-phenylindole (DAPI) revealed that only about 1% of *e2727* and *ok410* hermaphrodites carrying a rescuing array for ten generations contained chromosome fusions (*n* = 38 and 48, respectively), whereas 67 ± 10% of nonrescued *e2727* and *ok410* siblings displayed chromosome fusions (*n* = 30 and 40, respectively) (Figure 2C). Thus, the telomere replication defects of *e2727* and *ok410* were caused by mutation of the DY3.4 gene, which was therefore designated *trt-1 (telomerase reverse transcriptase-1).* Given that microinjection of *trt-1* mutants with linearized DNA fragments yielded extrachromosomal arrays that were used for rescue assays, formation of these transmissible genetic elements does not require telomerase activity, suggesting that they may be circular or that they may adopt a linear structure with noncanonical telomeres.

Taken together, the above results indicate that *e2727, yp1, tm899,* and *ok410* are alleles of *trt-1,* the C. elegans catalytic subunit of telomerase. Although our genetic evidence strongly suggests that *trt-1* is the C. elegans catalytic subunit of telomerase, confirmation that *trt-1* is required for telomerase activity in vitro will be important. However, such biochemical assays have yet to be established for *C. elegans,* despite significant efforts in this regard (L. Harrington, personal communication).

### *trt-1* Encodes a Compact TERT

**Figure 3:**
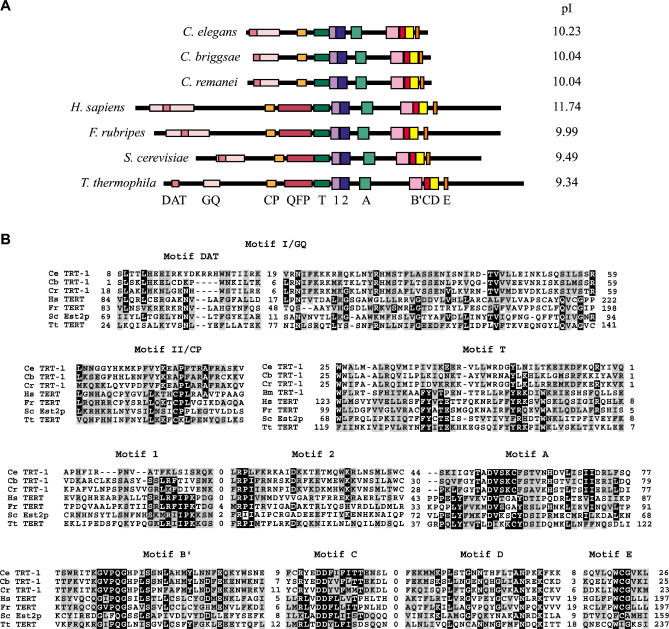
Structure and Sequence Motifs of *Caenorhabditis* TERT Proteins (A) Structure of TERT proteins from distantly related species, with isoelectric point (pI) indicated. (B) TERT sequence motifs include seven canonical reverse transcriptase motifs (1, 2, A, B′, C, D, and E) and several TERT-specific N-terminal motifs (I/DAT/GQ, II/CP, and T) [18–20] for TERT proteins from three *Caenorhabditis* species; *C. elegans (Ce), C. briggsae (Cb),* and *C. remanei (Cr);* from the vertebrates *H. sapiens (Hs)* and *Fugu rubripes (Fr);* and from *Saccharomyces cerevisiae (Sc)* and *Tetrahymena thermophila (Tt).* An additional motif T is included for the nematode *Brugia malayi (Bm),* as this sequence was critical for precisely defining the *Caenorhabditis* motif T. Only portions of motif I/GQ and motif DAT with homology to the *Caenorhabditis* TERT proteins are shown. Motif DAT is a specialized protein domain within motif I/GQ that is known to be required for telomerase activity in vivo [42]. A clearly defined III/QFP N-terminal motif was not identified for the *Caenorhabditis* TERTs. The Brugia malayi T motif is based on a short expressed sequence tag, and further N-terminal motifs could not be discerned from the current genome sequence (http://www.tigr.org). Sequence alignments were performed using Pole BioInformatique Lyonnais ClustalW 1.8 (http://pbil.ibcp.fr/htm/index.php).

*C. elegans trt-1* is predicted to encode only 540 amino acids and is one of the shortest TERT proteins known [16] (Figure 3A). The *trt-1* gene is tightly flanked by the neighboring genes DY3.3 and DY3.5 (http://www.wormbase.org), and RT-PCR analysis confirmed the predicted polyadenylation site at its 3′ end and that *trt-1* is *trans*-spliced to an SL2 spliced leader at its initiation methionine codon (unpublished data), as expected from whole genome analysis of SL2-spliced genes [17]. The domain structure of and a set of core amino acids in the seven canonical reverse transcriptase motifs found in all reverse transcriptases were sufficient to identify TRT-1 as a TERT protein [16] (Figure 3B). In addition, TERT proteins have a number of unique motifs that distinguish them from other reverse transcriptases [18–20], including motif T, which occurs immediately N-terminal to the canonical reverse transcriptase motifs. Conserved N-terminal TERT motifs were not recognizable for C. elegans TRT-1 using BLAST analysis, indicating significant evolutionary divergence [16]. However, comparison of the predicted TRT-1 polypeptide of C. elegans with those of the related nematodes C. briggsae and C. remanei revealed considerable N-terminal sequence homology (Figure S2A). When the N-termini of *Caenorhabditis* TRT-1 proteins were aligned with groups of TERT proteins from vertebrates, yeasts, or ciliates, homology with the DAT/I, GQ/I, and CP/II N-terminal motifs was apparent [18–20] (Figure 3, Figures S2B and S2C). In addition, an unusual motif T that lacked several highly conserved amino acids was observed for the *Caenorhabditis* genus, whose last common ancestor lived 100 million years ago. The more slowly evolving filarial parasitic nematode *Brugia malayi,* which diverged from C. elegans roughly 300 million years ago, had a motif T with greater similarity to those found in canonical TERT proteins (Figure 3B).

The last canonical reverse transcriptase motif of TERT, motif E, is typically followed by a telomerase-specific C-terminal domain of 150 to 200 amino acids, which harbors motifs that are essential for catalytic activity of telomerase in vitro and in vivo, for multimerization or processivity [21–23]. However, *Caenorhabditis* TRT-1 proteins had a C-terminal domain of less than 30 amino acids, which lacked substantial homology to any of the phylum-specific conserved C-terminal motifs found in other TERT proteins [16] (Figure 3 and unpublished data). Thus, *Caenorhabditis* TRT-1 has diverged substantially at both its N- and C-termini.

### *trt-1* and the *mrt-2* DNA Damage Checkpoint Gene Function in the Same Pathway

Mutations in the Ku heterodimer result in short but stable telomeres in Saccharomyces cerevisiae and Schizosaccharomyces pombe [24–27]. Yeast strains that contain mutations in both Ku and telomerase display faster telomere shortening and senescence phenotypes than do strains harboring a single telomerase mutation [25,27]. A synthetic phenotype between Ku and telomerase has also been reported for *Arabidopsis* [28]. Therefore, Ku is thought to affect telomere integrity via a telomerase-independent mechanism, perhaps by protecting telomeres from exonucleolytic attack [29]. In contrast, S. cerevisiae Mre11/Rad50/Xrs2 nuclease complex mutants have short but stable telomeres, but in this case double mutants defective for Mre11/Rad50/Xrs2 and the telomerase reverse transcriptase display the same telomere erosion and senescence phenotypes as telomerase mutants, arguing that these genes may function in the same telomere replication pathway [25,30].

**Figure 4:**
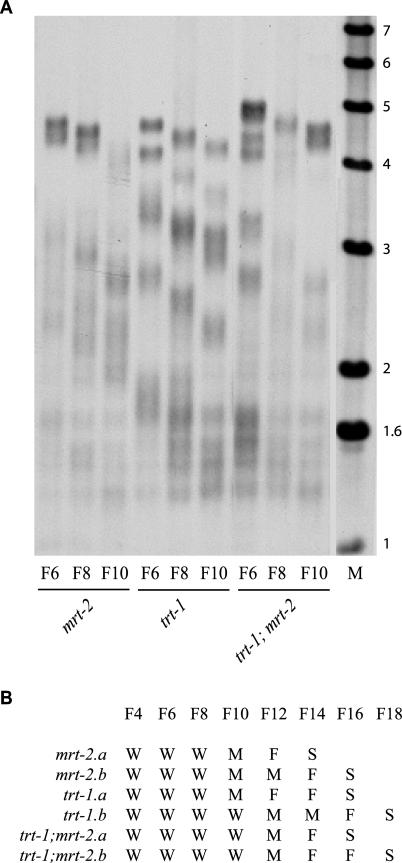
*trt-1* and *mrt-2* Function in the Same Pathway (A) Progressive telomere shortening of *mrt-2* single, *trt-1(tm899)* single, and *trt-1(tm899);mrt-2* double mutant strains. (B) Comparison of late onset of sterility in *mrt-2* single, *trt-1(tm899)* single and *trt-1(tm899);mrt-2* double mutant strains. Two independent strains, *a* and *b,* are shown for each genotype. Plates were seeded with six L1 animals, and the brood size was scored after 7 days of growth as wild-type (W), medium (M), few (F), and sterile (S) [12]. Note that initial telomere lengths observed for these strains reflect random telomere segregation during a cross. Therefore, the rate of telomere erosion is a more significant measure of additivity than are differences in initial telomere length or the time to sterility. Sizes of DNA fragments are indicated to the right (kb).

Null mutations in two subunits of the heterotrimeric 9–1–1 DNA damage response complex, *mrt-2* and *hus-1,* result in telomere erosion and late-onset end-to-end chromosome fusions in C. elegans [12,14], as observed for *trt-1* mutants (Figures 1 and 2). To determine if the 9–1–1 complex and *trt-1* act in the same pathway to facilitate telomere replication, rates of telomere erosion were measured for strains of *trt-1(tm899);mrt-2* double mutants and for *trt-1(tm899)* and *mrt-2* single mutants, all of which were derived from F2 siblings of the same F1 parent, thereby ensuring that their initial telomere lengths would be as similar as possible. Rates of telomere shortening in these strains were 135 ± 28 bp/generation for *trt-1(tm899),* 133.4 ± 34.7 bp/generation for *mrt-2,* and 119.4 ± 31.5 bp/generation for *trt-1(tm899);mrt-2* (Figure 4A; *n* = 2 independent strains each), which did not significantly differ from each other or from the rates observed for other *trt-1* alleles (Figures 2A and S1). In addition, the number of generations to sterility for both *trt-1(tm899)* and *mrt-2* single mutants and for *trt-1(tm899);mrt-2* double mutants was similar (Figure 4B). Identical results were observed with double mutant strains harboring mutations in *mrt-2* and either *trt-1(ok410)* or *trt-1(e2727)* (*n* = 2 for each *trt-1* allele, unpublished data). Thus, additivity was not observed for the rate of telomere erosion in *trt-1;mrt-2* double mutants, indicating that telomerase and the 9–1–1 checkpoint complex that responds to DNA double-strand breaks may act in the same telomere replication pathway. This conclusion is based on the premise that a rate of telomere shortening of greater than ~125 bp per generation is possible.

Although the 9–1–1 complex may be required for *trt-1* activity at chromosome termini, *trt-1* displays p53-mediated, ionizing radiation-induced apoptosis (B. Meier and S. Ahmed, unpublished data), whereas this response is abrogated in the *mrt-2* DNA damage response mutant [31]. Therefore, *trt-1* is not required for the 9–1–1 complex to respond to damage caused by ionizing radiation, such as DNA double-strand breaks.

### *trt-1* Mutants Become Sterile as a Consequence of Mitotic Failure

*S. cerevisiae ever shorter telomeres* mutants that are defective for telomere replication display a senescence phenotype in which the vast majority of cells die but “survivors” typically emerge that maintain their telomeres using homologous recombination [32]. Mammalian cells that lack telomerase can also survive telomere erosion by using a recombination-dependent process of telomere maintenance [33]. However, we have never observed survivors to arise for *C. elegans trt-1* mutants, despite having grown hundreds of *trt-1* strains to sterility (unpublished data). Thus, telomerase-independent recombination-based telomere maintenance may be incompatible with meiosis in *C. elegans,* a notion supported by the lack of any reported survivors in mouse strains deficient for telomerase [34].

Levels of apoptosis increase dramatically in the germlines of late-generation mouse telomerase RNA mutants [34], presumably as a consequence of telomere uncapping, end-to-end chromosome fusions, and/or subsequent breakage-fusion-bridge cycles. Telomere uncapping has also been shown to elicit either apoptosis or senescence in mammalian cells [35]. The C. elegans germline normally incurs a low background level of physiological apoptosis, and increased rates of apoptosis occur in response to either ionizing radiation or inactivation of *rad-51* [31,36], treatments that result in DNA double-strand breaks. However, levels of apoptosis were unaltered for all four *C. elegans trt-1* alleles in later generations, as compared to early generations or to wild-type controls (Table 1).

**Table 1:**
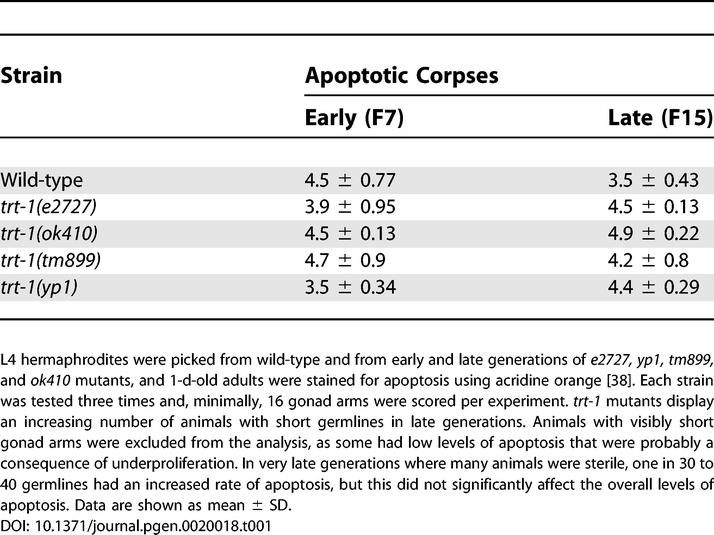
Basal Germline Apoptosis Is Normal in *trt-1* Mutants at Early (F7) and Late (F15) Generations

**Figure 5:**
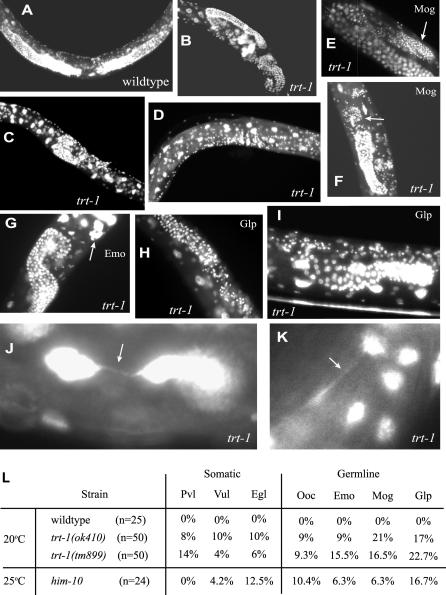
*trt-1* Mutants Display Mitotic Defects at Sterility (A) Wild-type germlines displayed two symmetric, highly proliferative arms of the germline when stained with DAPI. (B–K) In contrast, *trt-1* mutants at sterility displayed medium and small germline arms (B), small and empty germline arms (C), and only empty germline arms (D). Some *trt-1* mutants had a Masculinization of the Germline (Mog) phenotype and only produced sperm (E and F), many displayed endomitotic oocytes that endoreduplicate in the absence of sperm (G), some germlines displayed an abnormal Germline Proliferation (Glp) phenotype where the few germ cells that were produced were mitotic (H and I), and anaphase bridges were observed between intestinal nuclei in sterile *trt-1* adults (J and K) but not in wild-type controls. (L) Quantification of somatic and germline phenotypes in wild-type, in *trt-1* mutants, and in the kinetochore protein mutant *him-10* grown at the restrictive temperature of 25 °C [40]. Phenotypes scored were Protruding Vulva (Pvl), Vulvaless (Vul), Egg-Laying Defective (Egl), Intestinal Nuclear Bridge (Ibr), Abnormal Oocyte Formation (Ooc), Endomitotic Oocyte (Emo), Mog, and Glp. Although the Pvl phenotype was not observed in *him-10* animals, it is commonly observed for depletion of other kinetochore components by RNA interference (http://www.wormbase.org).

Given that late-generation *trt-1* mutants did not experience increased levels of apoptosis, sterile *trt-1* mutants were examined to assess the cause of their germline proliferation defects. *C. elegans rad-51* mutants display relatively normal levels of germ cell proliferation but lay hundreds of embryos that die as a consequence of aneuploidy and genome instability [37,38]. In contrast, few if any embryos were laid by *trt-1* hermaphrodites as they approached sterility, indicating that these mutants experience progressive impairment of germline proliferation (unpublished data). Several developmental defects were detectable in late-generation *trt-1* adults, such as a Protruding Vulva, a weak Uncoordinated phenotype, small worms with Body Morphology Defects, small sickly males, and occasional Slow Growth (unpublished data). A large percentage of *trt-1* mutant animals had short germline arms that ranged from medium size to an empty gonad (Figure 5A–5D). Gonad arm size often differed within a single animal, suggesting a stochastic germline proliferation defect, as might be expected for telomere replication mutants. High-resolution microscopy of sterile *trt-1* germlines revealed a variety of other defects, including masculinization of the germline, abnormal oocyte formation, endomitotic oocytes, and lack of sperm (Figure 5E–5I). In addition, chromatin bridges were observed between intestinal and embryonic nuclei of *trt-1* mutants but not between nuclei of wild-type controls (Figure 5J and 5K and unpublished data). Similar phenotypes have been reported previously for knockdown of *C. elegans* kinetochore components by mutation or RNA interference (http://www.wormbase.org) [39]. To confirm this possibility, we examined hermaphrodites defective for the *him-10* kinetochore gene [40], which incur high rates of mitotic chromosome nondisjunction. A spectrum of phenotypes similar to that found in sterile *trt-1* mutants was observed (Figure 5L), suggesting that *trt-1* mutants may become sterile as a consequence of chromosome segregation defects.

### Neither *trt-1* nor Telomere Length Affects C. elegans Life Span

**Figure 6:**
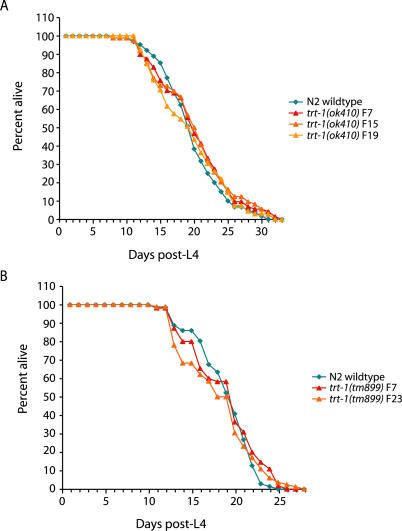
*trt-1* Mutants Have a Normal Life Span Independent of Telomere Length and Telomere Uncapping (A) Life spans of N2 wild-type and early, middle, and late generations of a *trt-1(ok410)* strain. Mutants were analyzed at generations with slightly shortened telomeres (F7), short telomeres (F15), and very short telomeres with chromosome fusions (F19). (B) Life spans of N2 wild-type and early and late generations of a *trt-1(tm899)* strain.

Many normal human somatic cells repress TERT expression and lack significant levels of telomerase activity [1,2], properties that result in telomere erosion and may contribute to aspects of the aging process. As mutation of *trt-1* ought to mimic the telomerase deficiency observed in human somatic cells, we sought to determine if either *trt-1* or telomere erosion might affect the aging process in C. elegans. Initial experiments were conducted with *trt-1(e2727)* or *trt-1(ok410)* strains, where early- and middle-generation strains were starved for several weeks prior to conducting life span analysis of early-, middle-, and late-generation strains in parallel. These studies suggested that neither *trt-1* nor telomere erosion affects life span in C. elegans (unpublished data). In order to control for any effects that extended starvation might have had on the early- and middle-generation samples described above, outcrossed strains homozygous for the *trt-1* deletion *ok410* were passaged, frozen at early-, middle-, and late-generations, and then thawed for life span analysis together. No change in life span was observed between wild-type (19.9 ± 0.6 days) and early- (20.6 ± 0.6 days), mid- (20.4 ± 0.6 days), or late- (20.0 ± 0.6 days) generations of a *trt-1(ok410)* strain (*P* = 0.18, 0.22, and 0.46, respectively), where telomeres of mid- and late-generation strains were about 1 and about 1.5 kilobases shorter than those of early-generation strains, respectively (Figure 6). Experiments with an additional allele of *trt-1* confirmed that life spans of N2 wild-type (19.1 ± 0.4 days) and early- (19.3 ± 0.5 days) or late- (18.3 ± 0.5 days) generations of a *trt-1(tm899)* strain were not significantly different (*P* = 0.96 and 0.23, respectively) (Figure 6). Similar results were obtained with independently derived *ok410* and *tm899* strains (Figure S3). The late-generation *trt-1* strains used for the above life span experiments displayed significant drops in brood size as a consequence of critically shortened telomeres being fused end-to-end (unpublished data). Thus, our results suggest that neither *trt-1* nor telomere erosion in the absence of *trt-1* nor chromosome uncapping and end-to-end fusion affects C. elegans life span.

## Discussion

Here we genetically define the C. elegans catalytic subunit of telomerase, *trt-1,* and study the effects of telomere erosion in *trt-1* mutants. TRT-1 is one of the smallest TERT proteins known as a consequence of truncated N- and C-terminal TERT-specific domains [16] (Figure 3). Functions known to be conferred by N- and C-termini of TERT include recruitment of telomerase to chromosome ends, nucleolar localization, and TERT multimerization [21–23]. Further, N-terminal domains of *Tetrahymena, S. cerevisiae,* and human TERT, including motif T, have been shown to mediate physical interactions with the telomerase RNA [18,41–43], so the divergent *Caenorhabditis* TERT N-terminus might reflect a telomerase RNA with unusual structural qualities. Consistent with this possibility, the C. elegans telomerase RNA has yet to be discovered in searches for noncoding RNAs with a telomeric template and secondary structures similar to those found in vertebrate telomerase RNAs [44], despite the availability of two fully sequenced *Caenorhabditis* genomes and extensive C. elegans cDNA sequencing [45,46]. Thus, TRT-1 has an unusual primary structure that may suggest functional divergence.

Mutation of yeast DNA damage checkpoint proteins typically results in shortened telomeres, whereas double mutants that lack both of the functionally redundant damage signaling proteins Mec1p/ATR and Tel1p/ATM have an Ever Shorter Telomeres phenotype that is similar to that observed for yeast telomerase mutants [47,48]. Thus, redundant DNA damage signaling is required for telomere replication in yeast. Since disruption of telomere structure by several means allows telomerase to act at telomeres in the absence of both Mec1p and Tel1p, the telomere-specific role of damage checkpoint signaling in yeast may be one of mediating telomerase access [49]. Two C. elegans orthologs of subunits of the 9–1–1 DNA damage response complex, *mrt-2* and *hus-1,* are required for telomere replication in C. elegans [12,14], whereas mutation in their S. cerevisiae or S. pombe orthologs results in short but stable telomeres [50,51]. We show here that *mrt-2* and *trt-1* may act in the same pathway (Figure 4), a possibility that may be difficult to assess in mammals, where the 9–1–1 complex is essential [52]. Given that the 9–1–1 complex can be loaded onto aberrant DNA structures at double-strand breaks and stalled replication forks [53], it may respond to replicating telomeres during S phase and assist with checkpoint-mediated modification or turnover of core telomere protein components. Thus, the redundant Mec1p/ATR and Tel1p/ATM checkpoint signaling pathways that function in yeast telomere replication may have been replaced by a single pathway in *C. elegans.* Alternatively, redundant checkpoint signaling may be required for mediating telomerase access in *C. elegans,* but the 9–1–1 complex may have an additional, essential role in facilitating telomerase activity.

In contrast to late-generation mouse telomerase RNA mutants [34], late-generation *trt-1* mutants failed to display an overall increase in apoptosis (Table 1), despite being proficient for p53-mediated, DNA damage-induced apoptosis (B. Meier and S. Ahmed, unpublished data). One explanation for this discrepancy might be that end-to-end chromosome fusions that result from telomere attrition in monocentric organisms such as mice and humans break frequently, which may elicit an apoptotic response. In contrast, C. elegans has holocentric chromosomes that can be propagated stably when fused, perhaps affording protection from break-induced apoptosis [54]. Although massive telomere uncapping can elicit an apoptotic response in mammalian cells [35], it is unclear how many DNA double-strand breaks are required to induce apoptosis in the C. elegans germline. Given that individual telomeres of a *trt-1* strain are likely to have different initial telomere lengths, only a small proportion of the 24 telomeres of a *C. elegans trt-1* mutant are likely to be uncapped at any point in time, even in later generations. Our data indicate that if uncapped telomeres are sensed as double-strand breaks in *C. elegans,* then the resulting DNA damage signaling is not sufficient to trigger apoptosis in *trt-1* mutants.

Levels of apoptosis were not enhanced in *C. elegans trt-1* mutants near sterility, but several phenotypes indicative of mitotic failure were observed (Figure 5). Given that *trt-1* mutants have typically incurred two or three end-to-end chromosome fusions by this time, subsequent fusion events may involve chromosome circularization. C. elegans has holocentric chromosomes that may interfere with mitotic segregation of circular chromosomes. Accordingly, the anaphase bridges observed between intestinal nuclei in late-generation *trt-1* mutants may reflect the presence of circularized chromosomes, which have never been recovered in C. elegans. Although circular chromosomes are mitotically stable in the monocentric yeast *S. pombe,* meiosis fails in the presence of such chromosomes [47]. Thus, chromosome circularization may result in mitotic as well as meiotic failure in late-generation *trt-1* mutants.

Linear end-to-end chromosome fusions consisting of two chromosomes typically display stable mitotic and meiotic transmission in C. elegans [12] (Figure 1A). In contrast, the only triple end-to-end fusion that has been constructed appears to break or mis-segregate frequently [55], and instability associated with multiply fused chromosomes may account for some of the phenotypes observed in late-generation *trt-1* mutants. For example, mitotic loss of a significant portion of the X chromosome during development of the germline could result in a Masculinization Of the Germline phenotype, which was displayed by sterile *trt-1* mutants and by mutants defective for chromosome segregation [39] (Figure 5). Other developmental phenotypes that occurred in sterile *trt-1* mutants, such as a Protruding Vulva, Uncoordinated neuromuscular defects, and occasional Slow Growth, have also been observed in C. elegans strains with chromosome segregation defects [39] (http://www.wormbase.org) (Figure 5). In summary, sterile *trt-1* mutants displayed a variety of phenotypes indicative of chromosome mis-segregation and/or mitotic failure, which may be the ultimate cause of sterility.

Some aspects of mammalian proliferative aging appear to be regulated by telomere erosion [6–10], but it is unclear if telomere length also affects postmitotic aging or whether the downregulation of TERT itself in human somatic cells might affect either proliferative or postmitotic aging by a mechanism that is independent of telomere length [56]. No difference in life span was observed between wild-type C. elegans strains and *trt-1* mutants with either long or short telomeres, even when critically shortened telomeres were present (Figures 6 and S3). These results suggest that neither telomere length nor TERT affects postmitotic aging of the soma in *C. elegans,* although proliferative immortality of the germline is clearly compromised. Thus, C. elegans TERT plays a role in proliferative rather than postmitotic aging. The presence of end-to-end fusions did not affect *trt-1* life span, but significant amounts of subtelomeric DNA are lost at the fusion breakpoints of some *C. elegans* end-to-end fusions (M. Lowden and S. Ahmed, unpublished data). Such sequence loss might occasionally compromise life span for some late-generation *trt-1* strains, although we failed to observe effects on life span in *trt-1* strains with multiple end-to-end fusions (Figures 6 and S3). A recent report suggests that modest increases in telomere length might extend postmitotic life span in C. elegans [57]. In this instance, it is possible that elongated telomeres correlated with extended life span but were not causal and that the observed effects on life span could have been due to another experimental variable. Indeed, a recent study of various wild strains of C. elegans failed to observe a correlation between significantly elongated telomeres and life span [58]. While this complements our observations that telomere shortening has no effect on life span for *trt-1* mutants, the relationship between elongated telomeres and life span in *C. elegans* will remain equivocal until strains with normal and significantly elongated telomeres are studied in isogenic backgrounds. We conclude that although telomere erosion in *C. elegans trt-1* mutants effectively mimics telomere dysfunction experienced by most human somatic cells, life span is not altered in these mutants. *trt-1* mutants may instead be useful for studying other properties of telomerase, such as its role in the development of cancer [59].

## Materials and Methods

### Strains and plasmids.

Strains were cultured at 20 °C as described [60], unless stated otherwise. The following mutant strains were used: *trt-1(e2727), trt-1(yp1), trt-1(tm899), trt-1(ok410), mrt-2(e2663), him-10(e1511), unc-13(e51)dpy-24(s71),* and *unc-29(e193). trt-1;mrt-2* double mutants were generated by crossing *unc-29;mrt-2* hermaphrodites with *trt-1* +/+ *unc-29* males. *mrt-2* F2 homozygotes were identified based on radiation hypersensitivity, whereas *trt-1* homozygotes were identified based on linkage with *unc-29.* The plasmid pSA1, which comprises the *trt-1* open reading frame as well as upstream and downstream sequences, was generated by digestion of cosmid H14N5 (Alan Coulson, personal communication) with EaeI and ligation into the NotI site of pBS-KSII^+^.

### RNA preparation and RT-PCR.

RNA was prepared with TriZOL (GIBCO-BRL, San Diego, California, United States). cDNA was generated from the RNA using SuperRT (HT Biotechnology Ltd, Cambridge, England) and an oligo(dT) linker [SKT: GCTCTAGAACTAGTGGATCCC(T)_20_]. To analyze *trt-1* transcription, PCR with primers DY15 (GCACCAACGATTAAGAGTTCAC) and DY16 (TTATTGCAACTTTATCAAAAATTGAC) was performed using cDNA obtained from N2 and mutant strains as a template. The spliced leader of *trt-1* mRNA was determined by PCR of N2 cDNA using either an SL1 (GGTTTAATTACCCAAGTTTGAG) or SL2 (GGTTTTAACCCAGTTACTCAAG) oligo as a forward primer and DY16.

### Rescue and complementation.

N2 genomic DNA, pCes1943 (gift from Diana Janke), and pSA1 (carrying the *trt-1* locus) were digested with PvuII, NruI, and ScaI, respectively. Mixtures of 100 ng of N2 genomic DNA, 2 ng of pCes1943, and 1 ng of pSA1 were injected into *trt-1(e2727)* and *trt-1(ok410)*. Rol and non-Rol siblings were propagated in parallel and scored for sterility. For noncomplementation analysis, *e2727, yp1,* or *tm899* was placed in *trans* to *unc-29* or *trt-1(ok410),unc-29,* and *trans*-heterozygotes were propagated by singling six non-Unc L4s each generation and selecting for the segregation of *unc-29*.

### Analysis of mutant phenotypes at sterility.

Fifty L4 larvae of each genotype were singled on NGM plates and scored for progeny after 48 h. Worms that produced progeny were passaged using one L4 per parent until a line became sterile (no progeny within 48 h post-L4). Sterile animals were stained with DAPI and scored for germline size, the presence of sperm and oocytes, the number of intestinal bridges, and chromosome number in oocytes.

### Life span analysis.

*trt-1(ok410)* strains were outcrossed numerous times to rejuvenate telomere length, and homozygous F2 lines were grown to sterility at 20 °C. Given that a variety of factors can affect C. elegans life span, it is best to perform analysis of different strains concurrently, under identical experimental conditions. For example, note the variation in maximum life span for the independent *ok410* and *tm899* experiments (Figures 6 and S3). Therefore, frozen stocks of *trt-1* mutants were created every four generations and then thawed and passaged on NGM plates for two generations prior to life span analysis. Each experiment was initiated with 100 L4 stage worms per genotype (six per plate). Animals were transferred every day for 1 week to separate them from their progeny and thereafter once a week. Worm viability was scored every day, and death was recorded when they failed to respond to light touch with a platinum pick. Worms that crawled off the plate and worms that died from an Egg Laying-Defective phenotype were excluded from the analysis. *P-*values were determined with the log-rank (Mantel-Cox) test using SAS software. Note that a recent study of telomere length and life span in C. elegans also relied on frozen stocks to examine life span for strains that were passaged for varying numbers of generations (Junho Lee, personal communication) [57].

## Supporting Information

Figure S1 Telomere Shortening Is Observed for All Four *trt-1* Alleles (3.0 MB TIF)

Figure S2 Additional TERT Multiple Sequence Alignments (1.9 MB TIF)

Figure S3 Life Span Is Normal for Both Early and Late Generations of Independent *trt-1(ok410)* and *trt-1(tm899)* Strains (1.6 MB TIF)

### Accession Numbers

The GenBank (http://www.ncbi.nlm.nih.gov/Genbank/index.html) accession numbers for the proteins shown in Figure 2 are CeTRT-1 (NP_492374), CbTRT-1 (CAE66970), CrTRT-1 (DQ178631), hTERT (AAG23289), fTERT (AAX59693), Est2p (AAB64520), TtTERT (AAC39140), and the Brugia malayi T-motif clone (AI658475).

## Abbreviations

bp: base pair
DAPI: 4′,6-diamidino-2-phenylindole
PCNA: proliferating cell nuclear antigen
TERT: telomerase reverse transcriptase
